# Voriconazole: a review of adjustment programs guided by therapeutic drug monitoring

**DOI:** 10.3389/fphar.2024.1439586

**Published:** 2024-12-06

**Authors:** Li Jiang, Zhiqiang Lin

**Affiliations:** Department of Pharmacy, Quanzhou First Hospital Affiliated to Fujian Medical University, Quanzhou, Fujian Province, China

**Keywords:** voriconazole, therapeutic drug monitoring, dose adjustment, individualized medication, concentration range

## Abstract

**Objectives:**

Exploring adjustments to the voriconazole dosing program based on therapeutic drug monitoring results to implement individualized therapy.

**Methods:**

PubMed and Embase were systematically searched to obtain study about voriconazole dose adjustment program guided by therapeutic drug monitoring. Quality evaluation and summarization of the obtained studies were performed to obtain program adjustments for voriconazole under therapeutic drug monitoring.

**Results:**

A total of 1,356 and 2,979 studies were searched on PubMed and Embase, respectively, and after removing irrelevant and duplicated studies, a total of 25 studies were included. A loading dose of 5 mg/kg q12 h or 200 mg q12 h and a maintenance dose of 50 mg q12 h or 100 mg q24 h is recommended for patients with Child-Pugh C. And in patients with Child-Pugh C, CYP2C19 genotype had no significant effect on voriconazole blood concentrations. Recommendations for presenting dosing programs based on different CYP2C19 genotypes are inconsistent, and genetic testing is not routinely recommended prior to dosing from a pharmacoeconomic perspective. Additionally, in adult patients, if the voriconazole trough concentration is subtherapeutic, the voriconazole dose should be increased by 25%∼50%. If the voriconazole trough concentration is supratherapeutic,the voriconazole dose should be decreased by 25%∼50%. If a drug-related adverse event occurs, hold 1 dose, decrease subsequent dose by 50%.In pediatric patients, if the voriconazole trough concentration is subtherapeutic, increase the voriconazole dose by 1∼2 mg/kg or increase the voriconazole dose by 50%. If the voriconazole trough concentration is supratherapeutic, reduce the voriconazole dose by 1 mg/kg or hold 1 dose, and decrease the subsequent dose by 25%.

**Conclusion:**

It is recommended that all patients on voriconazole should have their initial dosing program selected on the basis of their hepatic function or other influencing factors (e.g., pathogens, infections, C-reactive protein, albumin, or obesity), and that therapeutic concentrations should be achieved through appropriate dosage adjustments guided by therapeutic drug monitoring. Routine genetic testing for voriconazole application in patients is not considered necessary at this time. However, there has been a great deal of research and partial consensus on individualized dosing of voriconazole, but there are still some critical issues that have not been resolved.

## 1 Introduction

Voriconazole is a second-generation triazole antifungal drug with broad-spectrum antifungal activity, which is commonly used to treat invasive fungal disease in clinic, and is the first-line drug for invasive aspergillosis. The voriconazole trough concentration has been proved to be related to efficacy and toxicity, but there are still uncertainties in the process of voriconazole therapeutic drug monitoring (TDM) and individualized dosing (Perreault et al., 2019). Yi WM conducted the study in 151 adult patients, 68/151 (45.0%) of whom were critically ill. The study showed that voriconazole blood concentration monitoring is within the target concentration range (1.0∼5.5 mg/L) in only 134/250 (53.6%) of patients, <1.0 mg/L in 65/250 (26.0%), and >5.5 mg/L in 51/250 (20.4%), which suggests that voriconazole blood concentrations were not within the target concentration range in 116/250 (46.4%) of patients (Yi et al., 2017). Moreover, the probability that the trough concentration was within the target concentration range was increased two-fold compared to no dose adjustment when patients outside the target concentration range were dose-adjusted and the trough concentration was later reviewed (Yi et al., 2017). Sarah Perreault conducted a study in 128 adults with hematologic malignancies. The study showed that after 2 dose adjustments, 80% of patients were able to achieve the target concentration range (Perreault et al., 2019). One study based on pediatric patients (1.2∼18.5 years) showed that 55% of patients had voriconazole steady-state trough concentrations outside of the therapeutic concentration range, and 82% of these patients were able to achieve the therapeutic concentration range after dose adjustment (Lempers et al., 2019). Voriconazole exhibits nonlinear pharmacokinetics *in vivo* when administered at doses recommended in the drug insert for the treatment of invasive Aspergillus infections in adults or pediatrics. Numerous studies have been conducted on the individualized administration of voriconazole, but there are still some key unanswered questions regarding its clinical application. There are also current studies using Model-informed precision dosing (MIPD) to predict and optimize treatment outcomes based on patient characteristics and therapeutic drug monitoring data. In order to improve the clinical efficacy of voriconazole and to achieve individualized dosing of voriconazole, this study explored several aspects of the initial dosing program, the therapeutic concentration range, and the dose-adjustment program to provide a reference for obtaining the optimal clinical treatment program.

## 2 Materials and quality assessment

### 2.1 Data sources and searches

**FIGURE 1:**
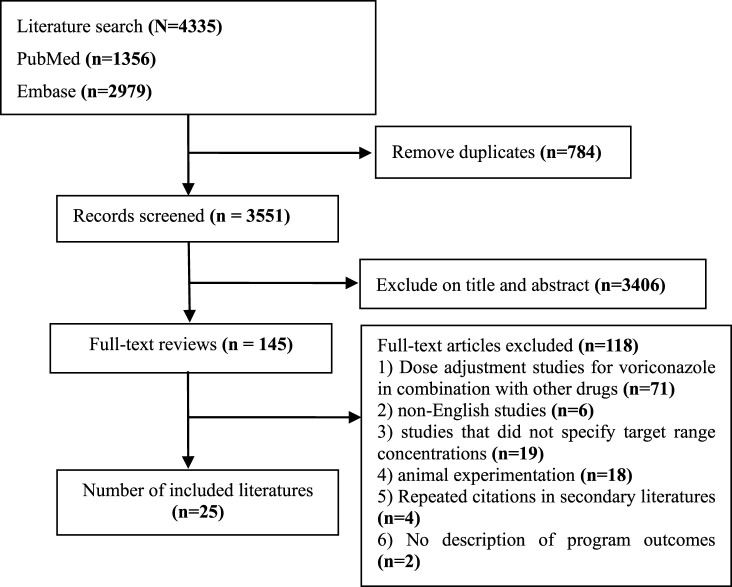
PRISMA flowchart.

The study searched the literatures for voriconazole administered under the guidance of therapeutic drug monitoring. The literatures covered program adjustments or made dose adjustments based on the patient’s liver function profile, genotyping, and body weight. Two researchers independently searched 2 databases (PubMed and Embase) from January 2002 to March 2024 to identify studies on voriconazole dose adjustment program guided by therapeutic drug monitoring (Figure 1). Once duplicates had been removed, the researchers identified studies eligible for analysis by examining titles and abstracts of every record, followed by full-text reviews. Any disagreement between reviewers was resolved by discussion, with arbitration by a third reviewer when required. The search strategy was:

(“voriconazole” OR “VRZ” OR “VRC” OR “VCZ”) AND (“dose adjustment” OR “dosage regimens” OR “dose modification”)
(“voriconazole” OR “VRZ” OR “VRC” OR “VCZ”) AND (“liver failure” OR “liver cirrhosis” OR “liver dysfunction”)
(“voriconazole” OR “VRZ” OR “VRC” OR “VCZ”) AND (“CYP2C19”)
(“voriconazole” OR “VRZ” OR “VRC” OR “VCZ”) AND (“obese” OR “obesity” OR “higher weight” OR “BMI” OR “body mass index”)
(“voriconazole” OR “VRZ” OR “VRC” OR “VCZ”) AND (“software”)

### 2.2 Inclusion and exclusion criteria

Inclusion criteria were decided according to PICOS. Participants (P): 1) patients were administered voriconazole, therapeutic drug monitoring was performed, and a target concentration range for voriconazole was specified, 2) inclusion of pediatric and adult populations. Intervention (I): propose an initial dosing program for special populations, or propose a dosage adjustment program based on a standard dosing program (For patients 12–14 years old and weighing >50 kg or patients ≥15 years old, the loading dose is 400 mg q12 h iv/po, and the maintenance dose is 200 mg q12 h iv/po. For patients aged 2–12 years old or younger, the loading dose is 9 mg/kg q12 h iv/po, and the maintenance dose is 8 mg/kg q12 h iv/po.). Control (C): The initial dosing program for special populations was compared with the standard dosing program, or dose-adjusted was compared with no dose adjustment. Outcome (O): Probability of target attainment (PTA). Study design (S): 1) human study, 2) inclusion of experimental studies (randomized controlled and non-randomized controlled studies), analytical studies (cohort and case-control studies) and pharmacokinetic modeling studies, 3) the study was available in English. Studies have also elaborated on the voriconazole guidelines.

The following studies were excluded: 1) dose adjustment studies for voriconazole in combination with other drugs, 2) studies that did not specify target range concentrations, 3) animal experimentation and laboratory study, 4) non-English studies.

Studies that excluded studies involving switching between different routes of administration were not performed but are labeled in the text.

### 2.3 Quality assessment

This study used the Newcastle-Ottawa Scale (NOS) (GA Wells et al., 2013) for quality assessment of case-control and cohort studies, MINORS (Slim et al., 2003) for quality assessment of non-randomized controlled interventional studies, and Jadad (Jadad et al., 1996) for quality assessment of randomized controlled interventional studies. For quality evaluation methods of pharmacokinetic modeling studies refer to Niu Wanjie et al. (2018).

The included studies are qualitatively described based on recommendations for initial dosing in specific populations, the implementation of TDM (target concentration ranges, dose recommendations following TDM, timing of repeat TDM), and MIPD.

After a literature search and quality assessment, the contents of the literature were categorized and discussed to derive recommendations for special populations (Child-Pugh C patients, patients with different CYP2C19 genotypes or other special populations) and dose adjustments.

## 3 Result

### 3.1 Literature search

A total of 1,356 and 2,979 studies were searched on PubMed and Embase, respectively, and after removing irrelevant and duplicated studies, a total of 25 studies were included in this paper, of which 4 studies were case-control studies, 4 were cohort studies, 5 were non-randomized controlled studies, 1 was a randomized controlled study and 11 used a modeling program, of which 9 were Population pharmacokinetics (Pop PK), and 3 were Physiologically based pharmacokinetics (PBPK). The study was also described for 5 voriconazole guidelines (Britain, Canada, China, Australia, Japan) (Ashbee et al., 2014; Laverdiere et al., 2014; Chen et al., 2018; Chau et al., 2021; Takesue et al., 2022).

### 3.2 Evaluation of the quality of literatures

Of the 25 studies, 4 studies (Zhou et al., 2020; Diller et al., 2021; Zembles et al., 2023; Zhang et al., 2023) were case-control studies and 4 (Yamada et al., 2018; Chaudhri et al., 2020; Tang et al., 2021; Zhao Y. C. et al., 2021) were cohort studies, all of which were evaluated for quality using the NOS (GA Wells et al., 2013), with 5 of them having 7“*” and 3 of them having 8“*”. 5 studies (Perreault et al., 2019; Gao et al., 2018; Hope et al., 2019; Hicks et al., 2020; Wang et al., 2021) were non-randomized controlled interventional studies and were evaluated for quality using MINORS (Slim et al., 2003), with four being moderate-quality literatures (scores of 13∼18) and two being high-quality literatures (scores of 19∼24). 1 study (Park et al., 2012) was a randomized controlled interventional study with quality assessment using Jadad (Jadad et al., 1996), which is a high-quality literature (scores of 4∼7). 11 studies (Pascual et al., 2012; Hope et al., 2013; Wang et al., 2014; Neely et al., 2015; Lin et al., 2018; Li et al., 2020; Li et al., 2021; Zubiaur et al., 2021; Jiang et al., 2022; Lin et al., 2022; Wang et al., 2024) applied the method of model simulation, and the quality evaluation method was referred to the study of Niu Wanjie et al. (2018). A total of 4 studies scored 77∼80, and a total of 7 studies scored 81∼86. Details are provided in Tables 1– 3.

**TABLE 1:** The newcastle-ottawa scale (NOS).

First Author(Year)	Case-control studies/Cohort studies	The Newcastle-Ottawa Scale			
		Selection	Comparability	Exposure/Outcome	NOS Score
Yamada et al. (2018)	Cohort studies	4	2	1	7
Chaudhri et al. (2020)	Cohort studies	4	2	1	7
Zhou et al. (2020)	Case-control studies	4	2	2	8
Diller et al. (2021)	Case-control studies	4	2	1	7
Zhao Y. C. et al. (2021)	Cohort studies	4	2	1	7
Tang et al. (2021)	Cohort studies	4	2	1	7
Zhang et al. (2023)	Case-control studies	4	2	2	8
Zembles et al. (2023)	Case-control studies	4	2	2	8

**TABLE 2:** MINORS and jadad.

First Author(Year)	Park et al. (2012)	Gao et al. (2018)	Perreault et al. (2019)	Hope et al. (2019)	Hicks et al. (2020)	Wang et al. (2021)
MINORS	No	Yes	Yes	Yes	Yes	Yes
1. A stated aim of the study		2	2	2	2	2
2. Inclusion of consecutive patients		2	2	2	2	2
3. Prospective collection of data		2	2	2	2	2
4. Endpoint appropriate to the study aim		2	2	2	2	2
5. Unbiased evaluation of endpoints		2	2	2	2	2
6. Follow-up period appropriate to the major endpoint		0	0	0	0	0
7. Loss to follow up not exceeding 5%		1	1	1	1	1
8. Prospective calculation of the sample size		0	0	0	0	0
And in the case of comparative studies						
9. A control group having the gold standard intervention		1	2	2	2	2
10. Contemporary groups		1	2	2	2	2
11. Baseline equivalence of groups		0	1	1	1	1
12. Statistical analyses adapted to the study design		1	2	2	2	2
MINORS score		14	18	18	18	18
Jadad	Yes	No	No	No	No	No
Randomized	2					
Random allocation	2					
Blinding	1					
Withdrawals and drop outs	1					
Jadad score	6					

**TABLE 3:** Model-related study scores.

First author(Year)	Title and abstract	Introduction	Methods	Results	Discussion and Conclusions	Others	Total scare
Pascual et al. (2012)	100	33	64	75	100	100	79
Hope et al. (2013)	86	66	56	75	100	100	81
Wang et al. (2014)	100	66	68	88	80	100	84
Neely et al. (2015)	86	66	60	75	100	100	81
Lin et al. (2018)	100	33	56	75	100	100	77
Li et al. (2020)	86	100	56	75	100	100	86
Zubiaur et al. (2021)	71	100	48	75	80	100	79
Lin et al. (2022)	100	67	68	75	100	100	85
Li et al. (2021)	86	100	80	75	100	50	82
Jiang et al. (2022)	86	33	84	75	100	100	80
Wang et al. (2024)	100	66	68	63	100	100	83

### 3.3 Initial dosing program for special populations

From 2013 to 2022, the relevant guidelines for drug monitoring for voriconazole therapy have been published or updated in Britain (Ashbee et al., 2014), Canada (Laverdiere et al., 2014), China (Chen et al., 2018), Australia (Chau et al., 2021) and Japan (Takesue et al., 2022), and many studies have also put forward suggestions on the initial dosage program for patients of different ages, Child-Pugh C, CYP2C19 genotype, race and Body Mass Index (BMI). Of the five guidelines, only the Britain and Japanese guidelines recommend an initial dosing program, with the British guideline recommending the same initial dosing program as the program in the specification (loading dose of 6 mg/kg q12 h iv or 400 mg q12 h po, and maintenance dose of 4 mg/kg q12 h iv or 200 mg q12 h po). The Japanese guidelines recommend the loading dose to be the same as the instructions, and the maintenance dose to be divided according to different populations and disease types. The maintenance dose is 4 mg/kg q12 h iv for non-Asian populations and 3 mg/kg q12 h iv for Asian populations. The maintenance dose for patients with *Candida* infections (except for *Candida* glabrata and *Candida* krusei) is 200 mg q12 h, and for patients with Aspergillus infections is 300 mg q12 h.

There were 7 studies on Child-Pugh C patients, 3 were prospectives and 4 were retrospectives, and of these 7 studies, 1 was conducted using the pop PK model, in which a loading dose of 5 mg/kg q12 h and a maintenance dose of 50 mg q12 h or 100 mg q24 h were recommended for Child-Pugh C patients (Lin et al., 2022). Of the remaining 6 studies, 2 studies suggested that the maintenance dose of voriconazole in Child-Pugh patients should be reduced to one-third of the standard dose (Zhang et al., 2023; Yamada et al., 2018). 2 studies recommended a loading dose of voriconazole of 200 mg q12 h (Gao et al., 2018; Wang et al., 2021), 1 study recommended 200 mg q24 h (Zhao Y. et al., 2021) and all three recommended a maintenance dose of 50 mg q12 h or 100 mg q24 h. In addition, 1 study was a stratified study using Total Bilirubin (TBIL) to determine the dosing program based on patients’ TBIL values (Tang et al., 2021). The specific studies are presented in Table 4.

**TABLE 4:** Initial dosing programs and target concentration ranges in patients with liver cirrhosis.

First author (Year)^4^	Country	Population^1^	Age/Male(N)	Weight	Number of patients	Initial dosing program	Target concentration range(mg/L)	Dosing program results
Gao et al. (2018)	China	Acute-on-chronic liver failure patients with development probably or diagnosis invasive pulmonary aspergillosis	42 (26∼70)/18	NR	20 (1 patients with cerebral failure, 9 patients with coagulation failure, and 2 patients with kidney failure)	The loading dose was 200 mg po q12 h. The maintenance dose was 100 mg po q.d	1.0∼5.0	Resulted in rational trough plasma drug concentrations (1.0∼5.5 mg/L), good clinical outcomes (90-day survival rate of 6/8) and no observed adverse events
Yamada et al. (2018)	Japan	Liver cirrhotic and non-liver cirrhotic patients	60(48∼64)/3	NR	6 patients with Child-Pugh C	The oral maintenance dose of voriconazole should be reduce to approximately one-third that of the standard dose	1.0∼5.0	Plasma voriconazole trough concentrations in all patients with Child-Pugh class C were almost within therapeutic range
Wang et al. (2021)	China	Patients with liver cirrhotic	50.5 ± 13.2 (22∼82)/95	63.1 ± 9.6 (40.5∼88)	120 (40 patients with Child-Pugh A/B and 80 patients with Child-Pugh C)^b^	The loading dose for Child-Pugh A/B patients was 200 mg q12 h with a maintenance dose of 75 mg q12 h or 150 mg q24 h, and the loading dose for Child-Pugh C patients was 200 mg q12 h with a maintenance dose of 50 mg q12 h or 100 mg q24 h	1.0∼5.0	The probability of the program achieving PTA at steady state (day 7) was 66.8%–72.3% for Child-Pugh A/B patients and 70.3%–74.0% for Child-Pugh C patients
Zhao Y. et al. (2021)	Asian	Patients with Child-Pugh C	49.35 ± 11.65 (32∼89)/39	61.27 ± 12.87 (36∼99)	43 (RMs with 1, NMs with 20, IMs with 16, PMs with 6)^c^	The loading dose was 200 mg q24 h, and the maintenance dose was 100 mg q24 h	1.0∼5.5	The target concentration range was achieved in 16 patients with initial dosing, 11 patients with 1 adjustment, 7 patients with 2 adjustments, and 9 patients with 3–9 adjustments
Tang et al. (2021)	China	Patients were diagnosed with liver cirrhotic	46.4 ± 12.8 (15∼89)/43	60.0 ± 13.1 (36∼99)	51 (4 patients with Child-Pugh A, 11 patients with Child-Pugh B, 36 patients with Child-Pugh C)	TBIL in the range of ULN to 51 μmol/L(TBIL-1), loading dose was 400 mg q12 h, followed by a maintenance dose of 100 mg q12 h iv/po TBIL in the range of 51–171 μmol/L(TBIL-2),A loading dose was 200 mg q12 h, followed by a maintenance dose of 50 mg q12 h or 100 mg qd iv/po TBIL ≥171 μmol/L(TBIL-3),A loading dose was 200 mg q12 h, followed by a maintenance dose of 50 mg qd iv/po	0.5∼5.0	The PTA for patients with TBIL-1 was 91.7% and 85.2%, administered orally and intravenously respectively The PTA for patients with TBIL-2 and TBIL-3 was highest(all>90%)
Lin et al. (2022)	China	Severe liver dysfunction	25(9∼31) /22	64.0 (47.5∼87.0)	26 (4 patients with Child-Pugh A, 8 patients with Child-Pugh B, 14 patients with Child-Pugh C	The loading dose for Child-Pugh A/B patients was 5 mg/kg q12 h with a maintenance dose of 100 mg q12 h or 200 mg q24 h, and the loading dose for Child-Pugh C patients was 5 mg/kg q12 h with a maintenance dose of 50 mg q12 h or 100 mg q24 h	2.0∼6.0	The PTA for patients with Child-Pugh A/B and C was 87.9% and 94.0%, respectively
Zhang et al. (2023)	China	Patients with Child-Pugh C	53.2 ± 13.4 /52	(BMI)23.1 ± 4.0 kg/m^2^	66 (NMs with 28, IMs with 25, PMs with 13)	The maintenance dose of voriconazole should be reduce to approximately one-third that of the standard dose	1.0∼5.5	Only 16.7% of patients with a trough concentration >5.5 mg/L and 4.5% with a trough concentration <1 mg/L had only one drug-related adverse event

^a^ All patients use voriconazole.

^b^ UMs, with 1; EMs, with 24; IMs, with 21; PMs, with 5.

^c^ RMs, with 12; IMs, with 11; PMs, with 3.

^d^ The starting dose route for (Zhang et al., 2023; Yamada et al., 2018; Gao et al., 2018) is oral administration. The starting dose route for (Lin et al., 2022) is intravenous administration. The starting dose route for (Tang et al., 2021; Wang et al., 2021) is intravenous or oral administration. The starting dose route for (Zhao Y. C. et al., 2021) is intravenous administration, oral administration or intravenous to oral sequential therapy.

q12 h, every 12 h q.d once daily; Iv, intravenously; Po, orally; TBIL, total bilirubin; PTA, Probability of target attainment; CYP2C19, cytochrome P2C19; UMs, ultrarapid metabolizers; RMs, rapid metabolizers; NMs, normal metabolizers; IMs, intermediate metabolizers; PMs, poor metabolizers; SCR, single-centre retrospective; SCP, single-centre prospective; MCR, multicentre retrospective; RCT, randomized controlled trial; ULN, upper limit of normal; BMI, body mass index; NR, no reference.

A total of 5 studies, 4 prospective and 1 retrospective, were conducted in patients with different CYP2C19 genotypes. The recommendations of these 5 studies varied, with only the patients with intermediate metabolizers (IM) being more consistent, with 4 studies (Hicks et al., 2020; Lin et al., 2018; Li et al., 2020; Zubiaur et al., 2021) suggesting that standard treatment programs could be used. Modeling methods were used in 4 of these 5 studies (2 for pop PK and 2 for PBPK).For patients with ultrarapid metabolizers (UMs), switching or using the program 500 mg q6 h for 48h, followed by 500 mg q8 h was recommended. For patients with rapid metabolizers (RMs), the maintenance dose was recommended to be 400 mg q12 h or using the program 400 mg q8 h for 24 h, followed by 500 mg q12 h. For patients with normal metabolizers (NMs) For patients with NMs, the recommended maintenance dose is 325–400 mg q12 h po or 200–300 mg q12 h iv or using program 400 mg q6h for 24 h, followed by 200 mg q12 h. For patients with IMs, 3 studies recommend using the standard dose, and 1 study suggests the maintenance dose of 275 mg q12 h or 175 mg t.i.d. For patients with poor metabolizers (PMs), 150∼200 mg q12 h iv or 225∼250 mg q12 h po is recommended, as well as the program 200 mg q12 h for 24 h, followed by 100 mg q24 h for a maximum of 2 weeks, followed by 50 mg q24 h for a maximum of 2 months. The remaining 1 study (Hicks et al., 2020) recommended that voriconazole be avoided in patients with the UMs phenotype, that the maintenance dose for patients with the RMs phenotype could be 300 mg bid po, and that patients with the remaining genotypes could be treated with the standard treatment program. These specific studies are listed in Table 5.

**TABLE 5:** Initial dosing program and target concentration range based on CYP2C19 genotype.

First author (Year)^b^	Country	Population^a^	Age/Male(N)	Weight (kg)	Number of patients	Initial dosing program	Target concentration range(mg/L)	Dosing program results
Lin et al. (2018)	China	patients with renal transplant recipients and different CYP2C19 genotypes	36(18∼58)/84	56.9 ± 10.5 (38.9∼87.5)	105(NMs with 44, IMs with 49, PMs with 12)	With NMs, the dosing program was 300 mg q12 h iv With IMs, the dosing program was 200 mg q12 h iv or 350 mg q12 h po With PMs, the dosing program was 150 mg q12 h iv or 250 mg q12 h po	2.0∼6.0	Patients with NMs, the PTA was 80.3% Patients with IMs, the PTA was 81.5% and 3.5% supratherapeutic concentrations Patients with PMs, the PTA was 90.9% and 6.3% supratherapeutic concentrations
Hicks et al. (2020)	America	patients with neutropenic AML and different CYP2C19 genotypes	64(19∼86)/139	80.4 (38.0∼165.8)	263(UMs with 5, RMs with 24, NMs with 105, IMs with 72, PMs with 7)^a^	With UMs, voriconazole is recommended to be avoided With RMs, the maintenance dose was 300 mg q12 h po With NMs, IMs, PMs, the standard dose program	The subtherapeutic trough concentration is < 1 mg/L	Subtherapeutic concentration were avoided in 83.8% of RMs receiving interventional dosage compared to 46.2% receiving standard dosage
Li et al. (2020)	Germany	Patients with different CYP2C19 genotypes	18∼53/NR	NR	305(RMs with 62, NMs with 101, IMs with 77, PMs with 65)	With RMs and NMs, the maintenance dose was 400 mg q12 h po With IMs, the standard dose program With PMs, no recommendations	1.5∼6.0	The dose program increased PTA two-fold while maintain a probability of reaching toxic concentration below 20%
Zubiaur et al. (2021)	Spanish	Patients with different CYP2C19 genotypes	23 (22∼25)/57	69 (68∼80)	106(UMs with 4, RMs with 33, NMs with 38, IMs with 29, PMs with 2)	With UMs, the dosing program was 500 mg q6h for 48 h,followed by 500 mg q8h With RMs, the dosing program was 400 mg q8h for 24 h,followed by 500 mg q12 h With NMs, the dosing program was 400 mg q6h for 24 h,followed by 200 mg q12 h With IMs, standard dosage or maintenance dose reduction to 100 mg q12 h (50% of the standard dose) With PMs, the dosing program was 200 mg q12 h for 24 h,followed by 100 mg q24 h for a maximum of 2 weeks, followed by 50 mg q24 h for a maximum of 2 months	0.5∼3.0	After dosage adjustment, the patient reached well tolerated and therapeutic voriconazole plasma levels
Li et al. (2021)	China	Patients with different CYP2C19 genotypes	37.5 ± 14.7/57	63.2 ± 12.3 (44.0∼111.0)	78 (NMs with 27, IMs with 32, PMs with 16)	With NMs, the dosing program was 325 mg q12 h or 200 mg t.i.d With IMs, the dosing program was 275 mg q12 h or 175 mg t.i.d With PMs, the dosing program was 225 mg q12 h or 150 mg t.i.d	2.0∼5.5	Patients with NMs, the PTA was 81.05% and 82.27% when the dosing program was 325 mg q12 h or 200 mg t.i.d respectively Patients with IMs, the PTA was 82.74% and 84.95% when the dosing program was 275 mg q12 h or 175 mg t.i.d respectively Patients with PMs, the PTA was 82.81% and 86.04% when the dosing program was 225 mg q12 h or 150 mg t.i.d respectively

^a^ Voriconazole was applied to 219 of 263 patients, with 202 receiving prophylactic voriconazole and 176 receiving a dose-adjustment program.

^b^ The starting dose route for (Hicks et al., 2020; Li et al., 2020; Li et al., 2021) is oral administration. The starting dose route for (Lin et al., 2018) is intravenous or oral administration. The starting dose pathway for (Zubiaur et al., 2021) is not mentioned.

AML, acute myeloid leukemia. q12 h, every 12 h q.d once daily. Iv, intravenously. Po, orally; TBIL, total bilirubin;; PTA, Probability of target attainment; CYP2C19, cytochrome P2C19; UMs, ultrarapid metabolizers; RMs, rapid metabolizers; NMs, normal metabolizers; IMs, intermediate metabolizers; PMs, poor metabolizers; ALB, albumin; SCR, single-centre retrospective; SCP, single-centre prospective; MCR, multicentre retrospective; RCT, randomized controlled trial; NR, no reference.

In addition, for overweight and obese patients, the Canadian guideline indicates that dosing based on true body weight (TBW) in obese patients (BMI ≥ 35 kg/m^2^) may increase the risk of overexposure and toxicity, and therefore recommends that intravenous and oral voriconazole dosing in obese patients should be based on ideal body weight (IBW) or adjusted body weight (AdjBW). The Japanese guidelines recommend the use of IBW or adjusted body weight dosing. It has also been suggested that dosing programs based on TBW are not appropriate for patients with a BMI ≥35 kg/m^2^, and the use of AdjBW for dosing calculation is recommended (Diller et al., 2021).

There are many factors that need to be considered in order to establish an individualized voriconazole dosing program, such as the type of infection, severity of infection, inflammation, BMI, and co-medication. The specifics of the 5 relevant studies are listed in Table 6, of which 3 were prospective studies, 2 were retrospective studies.

**TABLE 6:** Initial dosing program and target concentration range based on other factors^a^.

First author (Year)^d^	Country	Population^b^	Age/Male(N)	Weight (kg)	Number of patients	Initial dosing program	Target concentration range(mg/L)	Dosing program results
Pascual et al. (2012)	NR	Patients with Aspergillus and *Candida* infections	58(23∼78)/39	68(42∼125)	55 (27 patients with Aspergillus infection, and 8 patients with Candidiasis infection)	In acute infection, the dosing program was 300 mg iv and 400 mg po q12 h in responding infection or prophylaxis, the dosing program was 200 mg iv and 300 mg po q12 h	1.5∼4.5	The dosing programs of 300 mg iv and 400 mg po q12 h resulted in trough concentrations ≥1.5 in 87% and 78% of patients, respectively The dosing programs of 200 mg iv and 300 mg po q12 h resulted in trough concentrations ≥1.5 in 70% and 68% of patients, respectively
Wang et al. (2014)	China	Patients who diagnosed with a proven or probable IFIs	59 ± 21 (18∼99)/104	59.1 ± 7.8(35.0∼80.0)	151	with Aspergillus infections, the dosing program was 200 mg b. i.d iv/po with *candida* infections, the dosing program was 300 mg q12 h po or 200 mg q12 h iv	1.0∼4.0	For patients with Aspergillus infections, the CFR of the dosing program 200 mg q12 h iv and po was 95.8% and 94.2% respectively For patients with *Candida* infections, the CFR of the dosing program 300 mg q12 h iv and 200 mg q12 h po was 95.6% and 86.7% respectively
Diller et al. (2021)	America	Obese patients with BMI ≥35 kg/m^2^	TBW group was 61(44,70)/20 AdjBW group was 62(52,70)/47	TBW group was 87.1 (71.4, 96.7) AdjBW group was 96.2 (83.4, 109.1)	45 patients using TBW (Caucasian with 38, black with 5 and other with 2), 85 patients using AdjBW (Caucasian with 74, black with 8 and other with 3	AdjBW-based voriconazole dosing, combined with TDM, should be strongly considered in patients weighing ≥120% of their IBW.	1.0∼6.0	Therapeutic trough attainment was significantly improved with AdjBW-based dosing compared to TBW-based dosing (64.7% *versus* 46.7%; *p* = 0.047)
Jiang et al. (2022)	China	Patients with talaromy cosis	57(54∼69)/24 30(20∼65)/31	61(52∼72) 50(38∼87)	35 and 34 in the two hospitals respectively	CRP ≤ 96 mg/L, the loading dose was 250 mg q12 h, the maintenance dose was 100 mg q12 h CRP > 96 mg/L, the loading dose was 200 mg q12 h, the maintenance dose was 75 mg q12 h	1.0∼5.5	Patients with CRP ≤96 mg/L and CRP >96 mg/L had a 61.3% and 13.6% higher PTA with this optimal initial dosing program than the conventionally recommended program, respectively, and the potential for supratherapeutic concentrations decreased by 28.9%.^c^
Wang et al. (2024)	China	Elderly patients with hypoproteinemia	Modeling group was 70(60∼103)/70 Validation group was 79(65–93)/79	Modeling group was 63.00(36.1∼97.9) Validation group was 62.35(40.5∼100.0)	Modeling group had 128 patients Validation group had 22 patients	ALB≤35 g/L, the loading dose was 5 mg/kg q12 h, and the maintenance dose is 2 mg/kg q12 h iv ALB > 35 g/L,the loading dose was 5 mg/kg q12 h,and the maintenance dose is 3 mg/kg q12 h iv	2.0∼5.0	The loading dose of 5 mg/kg q12 h was adequate for patients with ALB≤35 g/L and ALB>35 g/L to attain high probabilities of target trough concentration range (94.18% and 90.68%) For patients with ALB ≤ 35 g/L, the probabilities of voriconazole reaching the target concentration range after a maintenance dose change was 99.26% and for patients with ALB > 35 g/L was 96.07%

^a^ Other factors are infection status, pathogens, body weight, C-reactive protein and albumin.

^b^ All patients use voriconazole.

^c^ The CRP, is an estimate of the proportion of the population achieving a target ∫AUC_24_/MIC, value ≥ 25, calculated by the PTAs, and the MIC, distribution of the microorganisms.

^d^ The starting dose pathway for (Diller et al., 2021; Jiang et al., 2022) is not mentioned. The starting dose route for (Wang et al., 2024) is intravenous administration. The starting dose route for (Pascual et al., 2012; Wang et al., 2014) is intravenous or oral administration.

q12 h, every 12 h q.d once daily; Iv, intravenously; Po, orally; PTA, Probability of target attainment; CYP2C19, cytochrome P2C19; UMs, ultrarapid metabolizers; RMs, rapid metabolizers; NMs, normal metabolizers; IMs, intermediate metabolizers; PMs, poor metabolizers; ALB, albumin; CFR, cumulative fraction of response; AdjBW, Adjusted body weight; TBW, total body weight; SCR, single-centre retrospective; SCP, single-centre prospective; MCR, multicentre retrospective; RCT, randomized controlled trial; CRP, C-reactive protein. NR, no reference.

### 3.4 Target trough concentration range

In addition to the target concentration ranges recommended by the five guidelines, 5 (Zhou et al., 2020; Lin et al., 2018; Li et al., 2021; Lin et al., 2022; Wang et al., 2024) studies chose different lower concentration limits and 8 (Diller et al., 2021; Zembles et al., 2023; Pascual et al., 2012; Lin et al., 2018; Li et al., 2020; Zubiaur et al., 2021; Lin et al., 2022; John et al., 2019) studies chose different upper concentration limits.

5 studies (Zhou et al., 2020; Lin et al., 2018; Li et al., 2021; Lin et al., 2022; Wang et al., 2024) used 2.0 mg/L as the lower limit of trough concentration. It is based on the study which included 34 patients and the results showed that none of the cases with trough concentration >2 mg/L were ineffective for voriconazole, while two-sixths of the cases with TDM below this threshold were nonresponsive (Dolton and McLachlan, 2014).

The upper limit of trough concentration also varied among studies. 1 study (Zubiaur et al., 2021) used 3.0 mg/L as the upper limit of trough concentration based on the meta-analysis studies. The results of meta-analysis study showed a significantly lower probability of hepatotoxicity at a trough concentration of <3.0 mg/L compared to the control group (RR = 0.37, 95% CI = 0.16∼0.83) (Jin et al., 2016). 1 study (Pascual et al., 2012) used 4.5 mg/L as the upper trough concentration limit, whose result shown that a >15% probability of neurotoxicity at trough concentrations >4.5 mg/L. Six studies (Diller et al., 2021; Zembles et al., 2023; Lin et al., 2018; Li et al., 2020; Lin et al., 2022; John et al., 2019) used 6.0 mg/L as the upper trough concentration limit based on the follow: Firstly, a meta-study consisting of 24 studies included literatures with >10 patients, TDM during voriconazole treatment, and studies that evaluated the relationship between trough concentration and clinical efficacy and/or safety. Literatures were searched from January 1998 through October 2013 and the following keywords were used: “voriconazole”, “triazole”, “vfend”, “drug monitoring”, “pharmacovigilance”, “adverse drug reaction/reporting system”. The meta-analysis showed that patients had an increased probability of adverse effects when trough concentrations ranged was 4.0∼6.0 mg/L (OR = 4.17,95 CI% = 2.08∼8.36) and that a supratherapeutic threshold of 6.0 mg/L was the most predictive of toxicity (OR = 4.60, 95% CI = 1.49–14.16) (Luong et al., 2016). The second is a study whose results show that elevated liver enzymes were frequently observed at voriconazole concentrations >6 mg/L, and this adverse event occurred in 8 of 11 cases at 6 mg/L or higher concentrations (Dolton and McLachlan, 2014). The target concentration ranges for each study are shown in Tables 4–8.

**TABLE 7:** Dose Adjustment program.

First author (Year)^a^	Population	Age/Male(N)	Weight (kg)	No. patients	Target through concentration range	Dose adjustment	Dose-adjusted outcome
Park et al. (2012)	South Korean	Adult/42	NR	55	1.0∼5.5 mg/L	<1.0 mg/L, increase dose by 100% >5.5 mg/L and there are no drug-related adverse events, decrease dose by 50% >10 mg/L or there are the drug-related adverse events happen, hold 1 dose, followed decrease dose by 50%	After subsequent dose modifications based on TDM, they were able to eventually reach the target in 75% of patients
Perreault et al. (2019)	Caucasian 78% Hispanic 12% Black 7% other 3%	Adult/72	NR^b^	128^c^	1.0∼4.0 mg/L	0.0∼0.6 mg/L, increase dose by 100 mg 0.7∼0.9 mg/L, increase dose by 50 mg 1.0∼4.0 mg/L, none 4.1∼5.5 mg/L, decrease dose by 50 mg 5.6∼7.9 mg/L, hold dose, recheck daily through concentration, then restart at 100 mg less when trough is ≤ 2.5 mg/L ≥8 mg/L, hold dose, recheck daily through concentration, then restart at 50% dose reduction when trough is ≤ 2.5 mg/L	After the second dose adjustment, they were able to eventually reach the target in 80% of patients Approximately 7.6% of patients developed an adverse effect with neurologic/psychological being the most common
Zhou et al. (2020)	Southeast Asians	Adult/113	75.0 (62.0∼85.0)	70 ^d^ (55 Chinese, 6 Indians, 5 Malaysians, 4 other)	2.0∼5.5 mg/L	<0.5 mg/L, re-load voriconazole at a dose of 1.5 times the new maintenance dose for 1 day, followed by 75% dose increase for maintenance dose (n = 1) 1.0∼1.9 mg/L, increase of 25%–33% (n = 6) and increase in 67% (n = 1) 5.5∼7.5 mg/L, reduction of 13% (n = 1) and reduction of 33% (n = 3) >7.5 mg/L, held off one dose or until neurological symptoms were resolved, followed by 33% reduction in dose (n = 6)	<0.5 mg/L, increased level by > 10 times(absolute increment is unknown as 0.5 mg/L was the upper limit of assay detection) 1.0∼1.9 mg/L, increased level by 70∼130% and increase level by 10% 5.5∼7.5 mg/L, reduction level by 50% and reduction level by 80% >7.5 mg/L, reduction level by >33% (absolute reduction is unknown as 7.5 mg/L was the upper limit of assay detection)
Zembles et al. (2023)	American	Pediatric/30	31.4 (14.1∼62.7)	59	1.0∼6.0 mg/L	<1.0 mg/L, increase dose by 50% >6.0 mg/L, hold 1 dose, decrease subsequent dose by 25%	After subsequent dose modifications based on TDM, they were able to eventually reach the target in over 80% of patients, though this required multiple stead-state voriconazole serum trough concentrations measurements

^a^ 3 patients were underweight (BMI < 18.50 kg/m2), 42 patients were normal weight (BMI, of 18.50∼24.99 kg/m2), 51 patients were overweight (BMI, of 25∼29.99 kg/m2), 27 patients were obese (BMI≥30 kg/m2) and 5 patients were morbidly obese (BMI ≥ 40 kg/m2).

^b^ A total of 250 monitoring sessions were performed in 128 patients, of which 237 were for preventive purposes (94.8%) and 13 for therapeutic purposes (5.2%).

^c^ One patient received voriconazole as anti-fungal prophylaxis whereas 62/70(88.6%) and 7/70(10%) patients were treated for IFIs, and CPA, respectively.

^d^ The starting dose route for (Perreault et al., 2019) is oral administration. The starting dose route for (Zhou et al., 2020) is intravenous or oral administration. The starting dose route for (Zembles et al., 2023) is intravenous or enteral administration. The starting dose route for (Park et al., 2012) is intravenous or oral or intravenous to oral sequential therapy.

SCR, single-centre retrospective; SCP, single-centre prospective; MCR, multicenter retrospective; RCT, randomized controlled trial; TDM, therapeutic drug monitoring; CAP, chronic pulmonary aspergillosis; BMI, body mass index; NR, no reference.

**TABLE 8:** Dose Adjustment program (Guideline).

First author (Year)	Country	Population	Target concentration range(mg/L)	Initial dosing program		Dose adjustment
				loading dose	Maintenance dose	
Ashbee et al. (2014)	Britain	All patients use voriconazole	1.0∼5.5 mg/L	6 mg/kg q12 h	4 mg/kg q12 h	<1.0 mg/L, increase dose by 50%,with a maximum dose of 6 mg/kg q12 h, or the oral voriconazole maintenance dose should be increased from 200 mg q12 h to 300 mg q12 h
Laverdiere et al. (2014)	Canada	All patients use voriconazole	prophylaxis ≥ 0.5 mg/L Treatment 1.5∼5.0 mg/L Toxicity < 5.5 mg/L	NR	NR	<0.5 mg/L, increase dose by 50% 0.5∼1.5 mg/L, increase dose by 25% 1.5∼5.5 mg/L, none ≥5.5 mg/L and drug-related toxicities, decrease dose by 25%
Chen et al. (2018)	China	All patients use voriconazole	0.5∼5.0 mg/L	NR	NR	<0.5 mg/L, increase dose by 50% 5.0∼10.0 mg/L without ≥ grade 2 adverse events, decrease dose by 20% >10.0 mg/L or has grade 2 adverse events, hold 1 dose, decrease subsequent dose by 50%
Chau et al. (2021)	Australia	Patient with haematological malignancy and haemopoietic stem cell transplant recipients	Prophylaxis and treatment 1.0–5.5 mg/L CNS infection, bulky disease, multifocal infection > 2 mg/L	NR	NR	0∼0.5 mg/L, increase dose by 50% 0.5∼1.0 mg/L, increase dose by 25% >5.5 mg/L and asymptomatic, decrease dose by 25% ≥5.5 mg/L and drug-related toxicities, hold 1 dose and decrease subsequent doses by 50%
Takesue et al. (2022)	Japan	Voriconazole in Asian and non-Asian populations	Asian 1.0∼5.5 mg/L Non-Asian 1.0∼4.0 mg/L	6 mg/kg q12 h	Asian 3 mg/kg q12 h Non-Asian 4 mg/kg q12 h	NR

q12 h, every 12 h. CNS, central nervous system; NR, no reference.

### 3.5 Dosage adjustment

A total of 4 dose-adjustment literatures were included in the study, 3 (Perreault et al., 2019; Zhou et al., 2020; Park et al., 2012) for adults and 1(15) for pediatrics. There is no consistently recommended program for dose adjustment of voriconazole. Of the 4 guidelines (Ashbee et al., 2014; Laverdiere et al., 2014; Chen et al., 2018; Chau et al., 2021) that propose a dosing program, details are given in Table 8.

In adult patients, 2 studies (Zhou et al., 2020; Park et al., 2012) recommended that if trough concentrations were much higher than supratherapeutic concentrations or if drug-related adverse events occurred, 1 dose should be reserved, subsequent doses should be reduced by 33% or 50%, and other antifungal drugs should be considered if toxicity was not reversed. In addition, the study by Sarah perreault et al. suggests that when trough concentration was 5.6∼7.9 mg/L, hold dose, and recheck daily through concentration, then restart at 100 mg less when trough concentration was ≤2.5 mg/L. When trough concentration ≥8 mg/L, hold dose, and recheck daily through concentration, then restart at 50% dose reduction when trough is ≤ 2.5 mg/L (PTA = 80%) (Perreault et al., 2019). 3 studies (Perreault et al., 2019; Zhou et al., 2020; Park et al., 2012) all recommended that the dose of voriconazole be reduced by 25% ∼ 50% if the trough concentration of voriconazole exceeded the therapeutic concentration and no drug-related adverse effects were observed. And sarah perreault et al. suggested that for trough concentration <0.5∼1.0 mg/L, voriconazole dose increase by 25%∼50%, which made 80% of patients were able to achieve the target concentration range with an adverse reaction rate of only 7.6% (Perreault et al., 2019).The study by WanBeom Park et al. recommended a 100% increase in voriconazole dose at trough concentrations <1 mg/L, with a dose-adjusted probability of target attainment (PTA) of 75% (Park et al., 2012). In addition to the above program, another program was proposed in the study by Pejun Yvonne Zhou et al. When the trough concentration was <0.5 mg/L, re-load voriconazole at a dose of 1.5 times the new maintenance dose for 24 h, followed by a 75% dose increase for the maintenance dose, which increased the level by > 10 times(absolute increment is unknown as 0.5 mg/L was the upper limit of assay detection). When the trough concentration was 1.0∼1.9 mg/L, the increase was 25∼33% or an increase of 67%, which increased the level by 70∼130% or increased the level by 10%. When the trough concentration was 5.5∼7.5 mg/L, reduction of 13% or reduction of 33%, which reduction level by 50% or reduction level by 80%. When the trough concentration was >7.5 mg/L, held off one dose or until neurological symptoms were resolved, followed by 33% reduction in dose, which reduction level by >33% (absolute reduction is unknown as 7.5 mg/L was the upper limit of assay detection) (Zhou et al., 2020).

There are fewer studies on dose adjustment for children and only 1 study by Zembles et al. (2023) was included in this study, which was studied in 59 pediatric patients aged 3.7∼14.7 years old. Of these, the study by Jamie john et al. did not further explore the post-therapeutic efficacy or PTA of dose adjustments, while the study by Tracy N. Zembles et al. showed that after subsequent dose modifications based on TDM, they were able to eventually reach the target in over 80% of patients, though this requires multiple steady-state voriconazole serum trough concentrations measurements. The specifics of the study are represented in Table 7.

### 3.6 Timing of repeat therapy drug monitoring

The timing of monitoring after application of the initial dosing program of voriconazole has become clearer, but there is still no clear evidence on the timing of re-monitoring after dose adjustment. WanBeom Park et al. argue that if the voriconazole dosage were or administration route was altered or if the interacting drug was introduced or halted, follow-up TDM was repeated on day 4 (Park et al., 2012). The British guideline recommends routine second monitoring to ensure that concentrations are stable and in the effective range, as well as repeat monitoring after dose adjustments and sequential therapy (Ashbee et al., 2014). The timing of repeat monitoring after dose adjustment is mentioned in the Chinese guideline, which thinks the timing of repeated therapy drug monitoring is consistent with the initial sampling time under the circumstance of no voriconazole loading dose, which is expected to be 4∼7 days after adjusting the voriconazole dosing program, or with initiating or withdrawing concomitant drugs that potentially influence voriconazole pharmacokinetic profiles (Chen et al., 2018). The Australian guidelines recommend that repeat testing should be on the fifth day after adjusting the dosing program, but no clear reason is given (Chau et al., 2021). Sarah Perreault et al. argued that trough concentrations should be rechecked on day 5 of the new dosing program (Perreault et al., 2019). Additionally, Tracy N. Zembles et al. suggested that steady state could be reached if voriconazole trough concentrations were repeated on ≥2.5 days after dose adjustment, but most studies chose to repeat monitoring on day 5 (Zembles et al., 2023).

With the exception of the Chinese guidelines, the remaining recommendations are expert opinions. The recommendations of the Chinese guidelines are based on the following 2 studies. Purkins L et al. evaluated the safety, tolerability, and pharmacokinetics of an intravenous to oral voriconazole program in 41 healthy males. The results showed that after switching from intravenous to oral dosing program, the majority of subjects reached a steady state on day 4, with the mean lowest trough concentration remaining above the clinically important Minimum Inhibitory Concentration (MIC) (Purkins et al., 2002). Visual inspection of trough concentration together with statistical analyses of peak concentration and Area under the curve (AUC) values suggest that steady-state levels were achieved by the 4–6 days of multiple dosing (Purkins et al., 2003).

### 3.7 Dose-prediction software

Several studies have used pharmacokinetic and pharmacodynamic modeling to accurately predict concentrations and dosages based on patient physiological data and drug concentration to guide rational clinical use. This approach is particularly useful for drugs with high pharmacokinetic variability, such as voriconazole. This study included four studies, 2 were software development studies (Hope et al., 2013; Neely et al., 2015) and two (Chaudhri et al., 2020; Hope et al., 2019) were software evaluations. Meanwhile, the two software development studies applied the Pop PK model, 1 of which was evaluated through prospective studies and the other through Monte Carlo simulations. In Table 9 are some of the software evaluations and related content.

**TABLE 9:** Dose-prediction software.

First author (Year)	study design	Software	Population	Number of patients	Advantages and disadvantages	Covariates
Software Development						
Hope et al. (2013)	SCP	Bestdose	HSCT recipients	10	Because parametric models are a single-model approach, they have no means to evaluate and optimize the expected precision with which a dosage regimen will achieve a desired target.	NR
Neely et al. (2015)	SCP	Cartride	Pediatrics under 18 years old	33	The control algorithm can accurately manage voriconazole therapy in children independently of steady-state conditions, and it is generalizable to any drug with a nonparametric pharmacokinetic model	Age, Weight
Software Evaluation						
Hope et al. (2019)	SCP	Bestdose	Patients with hematological malignancy or those undergoing HSCT	19	It is possible to achieve precise control for a compound with significant pharmacokinetic variability and non-linear PK. There are too few data in this study to enable the construction of new software that could utilise both genetype and voriconazole concentration	NR
Chaudhri et al. (2020)	SCR	DoseMeRx	Adult Australian patients	90^a^	Although the model assessed was developed in a Chinese population, the findings demonstrate that it is generalizable and can be extrapolated to other ethnicities	Total body weight, Height

^a^ A total of 110 surveillance sessions were performed in 90 patients, of which 41% were for prophylaxis and 59% for invasive fungal infections, with 86% of invasive fungal infections being Aspergillus infections.

SCR, single-centre retrospective; SCP, single-centre prospective; MCR, multicentre retrospective; RCT, randomized controlled trial; HSCT, hematopoietic stem cell transplantation; NR, no reference.

The names of the software developed in the two studies are Bestdose and Catrides. The Bestdose was created with data from 64 adults (20 healthy volunteers and 43 patients) and evaluated with pharmacokinetic data from 10 hematopoietic stem cell transplant (HSCT) patients. This program can be used to further optimize voriconazole for the treatment of critically immunocompromised patients, but still has many drawbacks for routine application (Hope et al., 2013). This prospective study has several limitations. Catrides, a nonparametric overall model containing 141 patients (85 children and 56 adults) and validated with 33 pediatric patients aged 8 months to 17 years old, showed that the advantages of the procedure are that patients do not have to be at steady state, sample sampling times do not have to be precisely timed, AUCs can be estimated even for a single concentration, and the procedure can be generalized to any drug with a nonparametric pharmacokinetic model, but prospective studies are still needed (Neely et al., 2015).

In a prospective clinical study evaluating Bestdose, results showed that 85.7% of patients had a trough concentration of 1.0–3.0 mg/L at 120 h after the start of treatment, which is above the 33% of the *a priori* expected proportion (Zembles et al., 2023). Kanika Chaudnri et al. included 90 patients to evaluate DoseMeRx and showed that dose prediction software enhances efficacy, is used to guide clinical decision-making, and can be generalized to other populations, although the model was developed in a Chinese population. However, the software did not monitor clinical outcomes and did not incorporate CYP2C19 genotypes (Chaudhri et al., 2020).

## 4 Discussion

### 4.1 Initial dosing program for special populations

There are accepted results for initial dosing programs, but dosing programs for special populations remain to be studied. The Japanese guidelines make different recommendations for maintenance doses for different diseases. The study was conducted by modeling Pop PK on data from 40 patients. The results suggest that transplant recipients receiving voriconazole for the prophylaxis of invasive candidiasis or aspergillosis may achieve target concentrations associated with the desired therapeutic outcome if the maintenance dose is 200 mg q12 h. However, Aspergillus with high minimal inhibitory concentrations may require higher maintenance doses (Perez-Pitarch et al., 2019).

For those with liver cirrhosis, trough concentrations of voriconazole can be affected by hepatic function due to the fact that voriconazole is primarily metabolized by the liver, and severe cholestasis significantly reduces voriconazole clearance, leading to slower metabolism and higher blood concentrations, which can increase the risk of drug-related adverse reactions, thus requiring clinical modification of the initial voriconazole dosing program (Pascual et al., 2012; Grensemann et al., 2021). The dosing program for Child-Pugh A/B is currently clearer, and the dosing program for Child-Pugh C needs further study. Of the 7 studies, 5 (Yamada et al., 2018; Tang et al., 2021; Gao et al., 2018; Wang et al., 2021; Lin et al., 2022) were unifactorial and 2 (Zhang et al., 2023; Zhao Y. et al., 2021) were multifactorial. In the 2 multifactorial studies, 1 study first investigated the influencing factors associated with variability in voriconazole trough concentration, performed multivariate bivariate correlation analyses, and developed multivariate linear regression models. The multiple regression linear model explained 34.8% of the variability in voriconazole trough concentration (R2 = 0.348), which implies that there is still a relatively high level of unexplained variability that requires further improvement. The results of another study showed that the mean trough concentration of patients with NMs, IMs, and PMs were 4.34 ± 2.12 mg/L, 4.40 ± 2.29 mg/L, and 4.30 ± 2.14 mg/L, respectively, and there was no significant difference between the three groups (*p* = 0.990), which ultimately suggests that only Child-Pugh classification affects trough concentration. Both of the 2 multifactorial studies explored the effect of CYP2C19 genotype in Child-Pugh C patients and both concluded that CYP2C19 genotype did not have a significant effect on trough concentration of voriconazole (*p* > 0.05).

And voriconazole is primarily metabolized in the liver by the CYP2C19 enzyme, with contributions by CYP2C9 and CYP3A4, and CYP2C19 polymorphisms could explain a substantial part of the remarkable inter-individual variability in voriconazole pharmacokinetics (Dean et al., 2012). Patients with CYP2C19 genotypes of IMs and PMs may be at higher risk of supratherapeutic levels and toxicity and the CYP2C19 genotyping as a potential strategy to optimize voriconazole dosing (Zhang et al., 2021). There have also been studies showing that poor metabolizers (most commonly in Asian populations) may experience higher voriconazole concentrations as well as a shift to other metabolic pathways for voriconazole such as 3A4 and 2C9 (McCreary et al., 2023).

It has been suggested that the level of inflammation (expressed as C-reactive protein concentration) can have an impact on voriconazole trough concentrations. Morgan et al. showed that inflammation stimulates the release of cytokines, leading to the regulation of hepatic transcription factor activity. These changes ultimately lead to the downregulation of most cytochrome P450 enzymes, affecting the production of metabolized proteins and thus reducing the clearance of certain drugs. In addition, *in vitro* studies have shown that pro-inflammatory cytokines, particularly interleukin-1 (IL-1), interleukin-6, and tumor necrosis factor alpha (TNF-α), downregulate the biosynthesis of a number of CYP450 enzymes involved in the metabolism of voriconazole, such as CYP2C9, CYP2C19, and CYP3A4 (Morgan, 2001; Aitken et al., 2006; Morgan et al., 2008). Van Wanrooy MJ et al. studied 128 patients and performed linear regression analyses of patient data unadjusted for covariates (sex, age, dose, route of administration, liver enzymes, and drug-drug interactions) and patient data adjusted for covariates. The results of the multiple linear regression analysis showed that for every 1 mg/L increase in C-reactive protein (CRP) concentration, voriconazole trough concentration increased by 0.015 mg/L (Encalada Ventura et al., 2015). Encalada Ventura MA et al. also correlated metabolic rate (MR) and CRP levels with voriconazole in 19 patients and showed a significant positive correlation between CRP and voriconazole concentration (rho = 0.62; 95% CI, 0.48 to 0.73; *p* < 0.001) and a negative correlation with MR (rho = −0.64; 95% CI, −0.77∼−0.50; *p* < 0.001), and voriconazole trough concentration increased with 0.021 mg/L for unit increase in the CRP level, and MR decreased with a −0.010 for every unit increase in the CRP level (*p* < 0.001 for both results) (Lee et al., 2021). In addition, although no dose adjustment is considered necessary for elderly patients in the voriconazole manual, it has been shown that elderly patients (age ≥ 65 years) have median voriconazole trough concentrations that are 80%–90% higher than those of younger patients after intravenous or oral voriconazole administration (Wang et al., 2014). This may be due to the fact that liver injury occurs in elderly patients and Cytochrome P450 levels decline after age 70 and result in an approximately 30% decrease in drug clearance (Stahl et al., 2018; Abdul-Aziz et al., 2020).

### 4.2 Target trough concentration range

Voriconazole demonstrates nonlinear saturation pharmacokinetics, resulting in unpredictable change in drug exposure (Abdul-Aziz et al., 2020; Gómez-López, 2020). AUC/MIC is the most predictive pharmacologic parameter, which >25 is closely related to clinical efficacy and patient survival against invasive fungal disease. The relationship between AUC and trough concentration of voriconazole has been demonstrated, and trough concentration/MIC can be used clinically in place of AUC/MIC (Troke et al., 2011; Carmo et al., 2023). Therefore, a defined range of target trough concentrations is of great importance for the clinical application of voriconazole therapy. The evidence for the target concentration ranges for each specific study is described above. In addition, there is evidence that the proportion of drug-resistant fungi has increased in recent years. It is hypothesized that the widespread use of antifungal drugs, the prolonged use of suboptimal concentrations, and the use of fungicides in agriculture have led to the development of genetic mutations that make fungi resistant to the drugs. The fungi most commonly associated with antifungal resistance are *Candida*, particularly non-albicans *Candida* and Aspergillus (Kolwijck et al., 2016; Huygens et al., 2023). And it has been shown that the MIC of drugs such as voriconazole has increased due to increased resistance to azoles, which can also have an impact on the target concentration range of voriconazole (Snelders et al., 2015; Pérez-Cantero et al., 2020). Therefore, this suggests that the proposed new target concentration range for voriconazole is meaningful.

There are also studies suggesting different target concentration ranges depending on the type of disease or population. For patients with Aspergillus infections, voriconazole trough concentration should be ≥ 2.0 mg/L. One study compared 107 first samples and 151 subsequent samples from 107 patients. Approximately one-third of the samples had voriconazole trough concentrations that deviated from the target concentration range and were mostly subtherapeutic. After predictive modeling, it was found that voriconazole used for the treatment of invasive aspergillosis had a higher probability of trough concentration >1.0 mg/L in subsequent samples than in first samples (*p* < 0.05) (Guinea et al., 2016). Another study tested four clinical wild-type and non-wild-type Aspergillus fumigatus isolates in an *in vivo* pharmacokinetic/pharmacodynamic model with voriconazole Clinical and Laboratory Standards Institute (CLSI) MICs ranging from 0.125 to 2.00 mg/L. By correlating trough levels with MICs, the study estimated that the highest MIC of an A. fumigatus isolate that may be treated successfully attaining the PK/PD target of EC_50_ and avoiding toxic serum trough levels of >5.5 mg/L albeit in a small proportion (<10%) of patients was 2, 4 and 1.5 mg/L for the CLSI, EUCAST and MTS methodologies, respectively. Moreover, considering that >90% of A. fumigatus isolates have CLSI MICs of ≤1 mg/L, the trough concentration of 2 mg/L will be required for PK/PD target attainment (Siopi et al., 2014). In addition, a target trough concentration ≥0.5 mg/L may be applicable for prophylaxis. Data on file at the United States Food and Drug Administration show that success rate in patients with fungal infections, whose mean voriconazole plasma levels were <0.5 g/mL, was 46% compared to 56% with mean plasma levels >0.5 g/mL (Trifilio et al., 2007).

### 4.3 Dosage adjustment

Four dose-adjustment-related studies (Perreault et al., 2019; Zhou et al., 2020; Zembles et al., 2023; Park et al., 2012) were included in this paper. Voriconazole dose adjustments guided by therapeutic drug monitoring are available in the British, Canadian, Chinese and Australian guidelines (Ashbee et al., 2014; Laverdiere et al., 2014; Chen et al., 2018; Chau et al., 2021). However, none of the four guidelines mentioned whether it was suitable for adults or pediatrics, and the British guideline did not mention the strength of evidence for the dose-adjustment program. The Canadian guideline stated that the dose-adjustment program was weak recommendation and low level of evidence and the Chinese guideline stated that the dose-adjustment program was conditional recommendation, very low quality of evidence. A dose-adjustment program was also suggested in the Australian guideline, but as the program was modified from the 2019 study by John et al. (2019), it was not included in the study to avoid duplication. Of the 4 studies other than guidelines, 3 studies explored voriconazole dose adjustment programs in adults, 1 study explored voriconazole dose adjustment programs in children.

Dose adjustment programs for adults were mentioned in 3 studies. WanBeom Park et al. conducted a randomized, evaluator-blinded, controlled, single-center study. The study was computerized and 108 patients were randomly assigned to either the TDM group (55 patients) or the non-TDM group (53 patients). The TDM group began blood collection on day 4 after voriconazole initiation and, based on the TDM results, adjusted the voriconazole dose according to the given dose adjustment program. dose to bring the trough concentration in line with the target concentration range. The non-TDM group maintained the standard dose of voriconazole. Ultimately, 27 patients in the TDM group had trough concentrations outside the target concentration range, six patients were not given dose adjustments due to discontinuation or death, and 21 patients received dose adjustments, of which 15 reached the target concentration range. The study still has limitations. First, the patients were only from a general hospital in Seoul, Korea, which was a single-center study, and the patients were all of Korean ethnicity. CYP2C19 test results showed that 43% of the patients were NM, 43% were IM, and 14% were PM, with a high percentage of poor metabolizers, which led to high levels of voriconazole concentrations in the study. Also, caution is needed when extrapolating this dose-adjustment program to other ethnic groups or pediatric populations whose pharmacokinetics different from those of adult patients. Second, the use of actual weight-based dosing in the study rather than fixed or ideal weight-based dosing may also have contributed to the high voriconazole levels. Third, the sample size was too small and larger sample sizes are still needed to determine the feasibility of the program (Park et al., 2012). Sarah perreault et al. conducted a prospective study with the primary objective of evaluating a voriconazole dose adjustment program. The study included 128 patients taking oral voriconazole for prophylaxis or treatment, of which 78% were Caucasian, 12% were Hispanic, 7% were black, 1% were Asian, and 2% were other populations. Of these 128 patients, 40% were overweight (BMI ≥ 25–29.99 kg/m^2^), 21% were obese (BMI ≥ 30 kg/m^2^), and 3.9% were morbidly obese (BMI ≥ 40 kg/m^2^). Dose adjustment was performed in these 128 patients, and 80% were able to achieve the target concentration range at the second dose adjustment. A subgroup analysis of patient-specific characteristics was also performed to obtain a higher percentage of patients >30 years old and BMI > 25 kg/m^2^ who initially reached the target concentration range, while age ≤30 years old and BMI ≤ 25 kg/m^2^ were mostly at subtherapeutic levels. The study still has limitations. First, the study was a single-center prospective study with a homogeneous patient population, most of whom were Caucasian and only one Asian, so extrapolation of the dose-adjustment program to other ethnic populations and pediatric populations needs to be done with caution. Second, only 32.8% of patients had a normal BMI, 2.3% had a BMI < 18.5 kg/m^2^, and 64.9% had a BMI ≥ 25 kg/m^2^, which is a disproportionately large number of patients with abnormal body weights who were administered a fixed dose. Finally, the study did not test the genotype of the patients and voriconazole was used for prophylaxis rather than treatment in 94.8% of patients (Perreault et al., 2019). A single-center retrospective study was also conducted by Pejun Yvonne Zhou et al. Seventy patients were included in the study, 55 of whom were Chinese, 5 Malays, 6 Indians, and 4 from other Asian ethnic groups. And 25 of the patients had probable or confirmed Aspergillus infection and 9 had probable or confirmed *Candida* infection. The study recommended dose adjustments based on the patients’ voriconazole trough concentration and obtained the effect on subsequent trough concentrations. In this study, only 45.7% of the patients achieved the target concentration range without dose adjustment. For patients with trough concentrations <0.5 mg/L, reloading voriconazole at a dose of 1.5 times the new maintenance dose for 1 day, followed by 75% dose increase for the maintenance dose (n = 1), which increased the level by > 10 times (absolute increment is unknown as 0.5 mg/L was the upper limit of assay detection). For patients with trough concentrations at 1.0∼1.9 mg/L, increasing the dose by 25∼33% (n = 6) and increase the dose by 67%(n = 1), which increased level by 70∼130% and increase level by 10%. For patients with trough concentrations at 5.5∼7.5 mg/L, reduction the dose by 13% (n = 1) and reduction the dose by 33% (n = 3), which reduction level by 50% and reduction level by 80%. For patients with trough concentrations >7.5 mg/L, held off one dose or until neurological symptoms were resolved, followed by 33% reduction in dose (n = 6),which reduction level by >33% absolute reduction is unknown as 7.5 mg/L was the upper limit of assay detection. There are several limitations to the study. First, the patients were all from Southeast Asian populations, 78.6% were Chinese, and only the adult population was included, so extrapolation of this dose adjustment program to other ethnic populations and pediatric populations still requires further study. Second, the study did not perform CYP2C19 genotype testing, so the effect of genetic polymorphisms on voriconazole trough concentration could not be determined. Finally, the sample size was too small, which resulted in the inability to elucidate the inhibitory or inducing effects of the various drugs and corresponding doses on CYP enzymes, and therefore further studies are still needed to recommend a voriconazole dose adjustment program (Zhou et al., 2020).

The incidence of invasive infections in children, although rare, is increasing with the rise of high-risk patients, including preterm infants, pediatric patients treated for hematologic malignancies, or allogeneic hematopoietic stem cell transplant recipients (Arrieta et al., 2023). In January 2019, the FDA expanded the indication for voriconazole to include children >2 years of age. Voriconazole pharmacokinetics is different and highly variable in pediatric patients compared to adults, and dosing is difficult, while oral bioavailability is lower (45%). Also, a dose-dependent pharmacokinetic profile of voriconazole was observed in the pediatric population, with a linear pharmacokinetic profile at voriconazole doses of 3∼4 mg/kg q12 h and a non-linear pharmacokinetic profile at 7∼8 mg/kg q12 h. Although the recommended dosage for children is given in the instructions, it has been shown that only 50% of pediatric patients achieve the target concentration at the first steady-state measurement and that children require a larger weight-based dose of voriconazole than adults to achieve the target concentration range, so pediatricians must often extrapolate voriconazole dosages for children from adult data (Zembles et al., 2023). In conclusion, it is challenging to optimize daily dosing to achieve the therapeutic range in pediatric patients (Arrieta et al., 2023; Resztak et al., 2021). A single-center retrospective study was conducted by Tracy N. Zembles et al. The study included 59 pediatric patients with a median age of 10.4 (3.7–14.7) years old. 42 patients had at least 1 measurement of steady-state trough concentration, 21 of whom were ≥12 years old and 21 of whom were <12 years old. Of these 42 patients, 13 patients (31%) had trough concentrations in the target concentration range at the first measurement, and after dose adjustment, 34 (81%) had trough concentrations in the target concentration range. The study included 7 years of longitudinal data collection, but shortcomings remain. First, the sample size was small. Patients were expected to be divided into <2 years old, 2–12 years old, and ≥12 years old subgroups, but because of the small number of patients <2 years old, they were divided into only two groups, <12 years old and ≥12 years old. Second, only 21 patients received a loading dose, which may have led to premature timing of voriconazole TDM in some patients who did not achieve steady-state blood levels. Finally, the study applied both intravenous and enteral administration, which may have resulted in lower voriconazole concentrations (Zembles et al., 2023). In conclusion, all current voriconazole dose-adjustment programs guided by TDM have significant limitations and still require further study and refinement.

### 4.4 Timing of repeat therapy drug monitoring

TDM should be repeated if the dose or route of administration of voriconazole is changed or if interacting drugs are introduced or discontinued, but the exact timing remains to be determined.

Purkins L et al. conducted 2 studies, one of which was a randomized, placebo-controlled, parallel-group, double-blind study of intravenous escalation and intravenous-to-oral switch study. The study divided 42 patients into 2 cohorts; 28 subjects were enrolled in Cohort 1 (14 on voriconazole, 14 on placebo) and 14 subjects were enrolled in Cohort 2 (7 on voriconazole, 7 on placebo). Patients in cohort 1 were treated with voriconazole 6 mg/kg q12 h iv for 24 h, followed by 3 mg/kg q12h, and then changed to an oral program of 200 mg q12 h po on days 8∼14. After 7 days of elution, switch to a higher maintenance dose (5 mg/kg q12 h iv, then change to an oral program 400 mg q12 h) Cohort 2 used a program of 4 mg/kg q12 h iv, followed by a switch to an oral program of 300 mg q12 h po. The results showed that after switching from intravenous to an oral dosing program, the majority of subjects reached a steady state on day 4, with the mean lowest trough concentration remaining above the clinically important MIC (Purkins et al., 2002). Another study is a single-blind, multiple-dose, placebo-controlled, parallel-group, dose-finding study. The study divided 64 patients into groups of 8 subjects each receiving voriconazole doses of 2 mg/kg q12 h, 4 mg/kg q12 h, 2 mg/kg t.i.d or 3 mg/kg q12 h. Eleven subjects received 1.5 mg/kg t.i.d, and 21 patients received placebo. The study demonstrated by statistical analysis of peak concentrations and area under the plasma concentration-time curve (AUCτ) from just before dosing to the end of the dosing interval, as well as visual inspection of trough concentrations, that steady state levels were reached on the third or fifth day of multiple dosing (Purkins et al., 2003). However, the former study included only 41 patients and the latter study included only 56 patients. Neither of these two papers were up-to-date. In the future, researchers should pay more attention to the timing of repeat TDM with voriconazole and study it further.

### 4.5 Dose-prediction software

MIPD is a mathematical modeling and simulation technology that integrates information about the patient, drug, and disease to provide a basis for precise patient dosing. The MIPD often uses nonlinear mixed-effects (NLME) models to predict and optimize treatment outcomes based on patient characteristics and therapeutic drug monitoring data. MIPD is indicated for drugs with narrow therapeutic ranges and complex pharmacokinetics (PK), such as voriconazole (Kluwe et al., 2023). Commonly used models include, but are not limited to, population pharmacokinetic (Pop-PK) models, pharmacokinetic/pharmacodynamic (PK/PD) models, population pharmacokinetic/pharmacodynamic models, physiologically based pharmacokinetic (PBRK) models and artificial intelligence (AI) models, and different modeling and analysis techniques have different characteristics. Voriconazole dose prediction procedures are mostly based on Pop-PK models. Such models should at a minimum contain the most relevant physiological and biological attributes determining the drug’s disposition and enough attributes to explain a substantial portion of observed variability. A pop-PK model can be validated internally, externally, or prospectively to diagnose misspecifications (Darwich et al., 2021; Wicha et al., 2021).

Bestdose was created with data from 64 adults (20 healthy volunteers and 43 patients) and evaluated with pharmacokinetic data from 10 hematopoietic stem cell transplant (HSCT) patients. Validation of the performance of the voriconazole controller showed close agreement between the controller-calculated voriconazole dose and the actual dose administered in most cases, but in two patients the controller predicted significantly more medication than was actually administered. This program can be used to further optimize voriconazole for the treatment of critically immunocompromised patients, but still has many drawbacks for routine application (Hope et al., 2013). The procedure needs to be evaluated for accuracy in prospective clinical trials, and the applicability of oral voriconazole to the procedure needs to be tested, as well as other existential issues. In a prospective clinical study of Bestdose, 19 patients (18 Caucasian, 1 other) were included for evaluation of the procedure, but only 14 could be analyzed. 12 of the 14 patients (85.7%; 95% CI, 57.2%–98.2%) had a trough concentration of 1.0∼3.0 mg/L at 120 h after the start of treatment, which is above the 33% of the *a priori* expected proportion (Hope et al., 2019). This prospective study has several limitations. The sample size of the study was relatively small, containing only patients in the early stages of HSCT rather than those with critical disease leading to potentially more variable and extreme pharmacokinetics, and the duration of the study was relatively short, leaving many issues unexposed. Cartrides, a nonparametric overall model containing 141 patients (85 children and 56 adults) and validated with 33 pediatric patients aged 8 months to 17 years old, showed that the advantages of the procedure are that patients do not have to be at steady state, sample sampling times do not have to be precisely timed, AUCs can be estimated even for a single concentration, and the procedure can be generalized to any drug with a nonparametric pharmacokinetic model, but prospective studies are still needed (Neely et al., 2015). Kanika Chaudnri et al. included 90 patients to evaluate DoseMeRx and showed that dose prediction software enhances efficacy, is used to guide clinical decision-making, and can be generalized to other populations, although the model was developed in a Chinese population. However, the software did not monitor clinical outcomes and did not incorporate CYP2C19 genotypes (Chaudhri et al., 2020).

The study summarized the initial treatment program, target concentration range, and dose adjustment program for voriconazole and identified the following shortcomings in the study. 1) the included studies did not have the same target concentration range, making it difficult to compare studies with the same factors; 2) some of the studies used modeling methods that were not implemented in the clinic, resulting in uncertainty about the clinical efficacy of the programs; and 3) the inclusion of fewer literatures related to dosage adjustments, which made it difficult to draw accurate conclusions.

## 5 Conclusion

There has been a great deal of research and partial consensus on individualized dosing of voriconazole, but there are still some critical issues that have not been resolved. Recently updated guidelines for TDM of voriconazole or antifungals focus on key issues that need to be addressed. The 2021 edition of the Australian guideline focuses on 9 issues regarding TDM of antifungals. The 2022 edition of the Japanese guideline focuses on 5 issues regarding TDM of voriconazole. There is still no clear and uniform program for dose adjustment of voriconazole guided by TDM. Based on the results of the study, it is recommended that all patients on voriconazole should have their initial dosing program selected on the basis of their hepatic function or other influencing factors (e.g., pathogens, infections, C-reactive protein, albumin, or obesity), and that therapeutic concentrations should be achieved through appropriate dosage adjustments guided by therapeutic drug monitoring. Routine genetic testing for voriconazole application in patients is not considered necessary at this time. In terms of dose adjustment, in adult patients, if the voriconazole trough concentration is subtherapeutic, the voriconazole dose should be increased by 25%∼50%. If the voriconazole trough concentration is supratherapeutic,the voriconazole dose should be decreased by 25%∼50%. If a drug-related adverse event occurs, hold 1 dose, decrease subsequent dose by 50%.In pediatric patients, if the voriconazole trough concentration is subtherapeutic, increase the voriconazole dose by 1∼2 mg/kg or increase the voriconazole dose by 50%. If the voriconazole trough concentration is supratherapeutic, reduce the voriconazole dose by 1 mg/kg or hold 1 dose, and decrease the subsequent dose by 25%. Most of the previous clinical studies have a low level of evidence-based medicine evidence, and more prospective, multicenter clinical studies are needed to promote individualized dosing of voriconazole.

## Abbreviations

q12 h, every 12 h; q.d, once daily; Iv, intravenously; Po, orally; TBIL, total bilirubin; PTA, Probability of target attainment; CYP2C19, cytochrome P2C19; UMs, ultrarapid metabolizers; RMs, rapid metabolizers; NMs, normal metabolizers; IMs, intermediate metabolizers; PMs, poor metabolizers; SCR, single-centre retrospective; SCP, single-centre prospective; MCR, multicentre retrospective; RCT, randomized controlled trial; ULN, upper limit of normal; AML, acute myeloid leukemia; ALB, albumin; CFR, cumulative fraction of response; AdjBW, adjusted body weight; TBW, total body weight; CRP, c-reactive protein; TDM, therapeutic drug monitoring; CAP, chronic pulmonary aspergillosis; HSCT, hematopoietic stem cell transplantation; NR, no reference; MIPD, model-informed precision dosing; NLME, nonlinear mixed-effects; CNS, central nervous system.
